# Comparison and analysis of reoperations in two different treatment protocols for trochanteric hip fractures – postoperative technical complications with dynamic hip screw, intramedullary nail and Medoff sliding plate

**DOI:** 10.1186/s12891-017-1723-x

**Published:** 2017-08-24

**Authors:** Johnny Paulsson, Josefine Corin Stig, Ola Olsson

**Affiliations:** Department of Orthopedics, Helsingborg Hospital, S-251 87 Helsingborg, Sweden

**Keywords:** Pertrochanteric, Subtrochanteric, Fracture, Reoperation, Intramedullary nail, Medoff sliding plate, Dynamic hip screw

## Abstract

**Background:**

In treatment of unstable trochanteric fractures dynamic hip screw and Medoff sliding plate devices are designed to allow secondary fracture impaction, whereas intramedullary nails aim to maintain fracture alignment. Different treatment protocols are used by two similar Swedish regional emergency care hospitals. Dynamic hip screw is used for fractures considered as stable within the respective treatment protocol, whereas one treatment protocol (Medoff sliding plate/dynamic hip screw) uses biaxial Medoff sliding plate for unstable pertrochanteric fractures and uniaxial Medoff sliding plate for subtrochanteric fractures, the second (intramedullary nail/dynamic hip screw) uses intramedullary nail for subtrochanteric fractures and for pertrochanteric fractures with intertrochanteric comminution or subtrochanteric extension. All orthopedic surgeries are registered in a regional database.

**Methods:**

All consecutive trochanteric fracture operations during 2011–2012 (*n* = 856) and subsequent technical reoperations (*n* = 40) were derived from the database. Reoperations were analysed and classified into the categories adjustment (percutaneous removal of the locking screw of the Medoff sliding plate or the intramedullary nail, followed by fracture healing) or minor, intermediate (reosteosynthesis) or major (hip joint replacement, Girdlestone or persistent nonunion) technical complications.

**Results:**

The relative risk of intermediate or major technical complications was 4.2 (1.2–14) times higher in unstable pertrochanteric fractures and 4.6 (1.1–19) times higher in subtrochanteric fractures with treatment protocol: intramedullary nail/dynamic hip screw, compared to treatment protocol: Medoff sliding plate/dynamic hip screw. Overall rates of intermediate and major technical complications in unstable pertrochanteric and subtrochanteric fractures were with biaxial Medoff sliding plate 0.68%, with uniaxial Medoff sliding plate 1.4%, with dynamic hip screw 3.4% and with intramedullary nail 7.2%.

**Conclusions:**

The treatment protocol based on use of biaxial Medoff sliding plate for unstable pertrochanteric and uniaxial Medoff sliding plate for subtrochanteric fractures reduced the risk of severe technical complications compared to using the treatment protocol based on dynamic hip screw and intramedullary nail.

## Background

Stable two-fragment pertrochanteric fractures are treated with good result with dynamic hip screw (DHS) devices [1, 2], but controversy remains regarding treatment of unstable pertrochanteric and subtrochanteric fractures. Dynamic fixation methods are designed to allow secondary fracture impaction to improve interfragmentary stress transfer, thereby facilitating fracture healing and unloading the implant. DHS allows dynamic compression along the axis of the femoral neck, which, depending on fracture geometry, may be more or less appropriate or effective [3, 4].

The Medoff sliding plate [5] (MSP) adds compression along the axis of the femoral shaft, thus allowing biaxial dynamisation. Lag screw sliding is optional and may be blocked by a locking screw to prevent medialisation of the femoral shaft, thereby creating uniaxial dynamisation.

Intramedullary nail (IMN) fixation is most commonly used worldwide for unstable pertrochanteric and subtrochanteric fractures and provides a strong and rigid fixation [6]. However, poor load transfer may subject the implant to high loads with increased risk of nonunion, implant failure or fracture due to stress concentration [2, 7].

In Skåne, Sweden, two similar regional emergency care hospitals use different treatment protocols for trochanteric fractures. For pertrochanteric fractures considered as stable both hospitals use DHS, whereas for fractures considered as unstable within the respective treatment protocol, Helsingborg Hospital uses MSP, and Kristianstad Hospital uses IMN. All orthopedic operations performed are consecutively registered in a mutual regional database and both hospitals cover similar population based catchment areas.

The aim of this consecutive retrospective study was to analyse and compare all performed reoperations and related technical complications in all pertrochanteric and subtrochanteric fractures, treated during two years according to two different treatment protocols in two parallel consecutive case series.

## Methods

The two treatment protocols for choice of fixation methods (MSP/DHS or IMN/DHS) are described in Table 1. In short, DHS was used in fractures considered as stable, whereas treatment protocol: MSP/DHS aimed to use MSP in all pertrochanteric fractures with 3 or more fragments and treatment protocol: IMN/DHS aimed to use IMN in fractures with intertrochanteric comminution or fracture extension below the trochanters. In subtrochanteric fractures treatment protocol: MSP/DHS used locked MSP and treatment protocol: IMN/DHS used IMN. The implants used were the Medoff sliding plate (MSP) or Hansson plate (DHS) (Swemac Orthopaedics, Linköping, Sweden), or IM nails (Gamma Nail (Stryker, Kalamazoo, MI, USA), Intertan and Trochanteric Anterior Nail (Smith&Nephew, Memphis, TN, USA). The DHS and MSP were combined with either a Twin hook [8] (*n* = 697) (Swemac, Orthopaedics, Linköping, Sweden) or a lag screw (*n* = 5). The Twin hook wings may be retracted, which allowed percutaneous technique with the plate left in situ when adjustment, replacement or removal of the Twin hook was needed. The MSP was used in biaxial or uniaxial mode (locking screw for the Twin hook/lag screw). In treatment protocol: MSP/DHS repeat radiographs were obtained 10 days postoperatively when MSP was used in uniaxial mode. If axial sliding capacity of the plate was consumed (25 mm), or migration of the Twin hook/lag screw was observed, the locking screw was routinely removed to allow biaxial dynamisation.

**Table 1:** Treatment protocols for pertrochanteric and subtrochanteric fractures in the two treatment groups

Fracture type		Fixation method	
		Treatment protocol: MSP/DHS^a^	Treatment protocol: IMN/DHS^b^
Pertrochanteric			
	Stable - 2 fragments^c^	DHS	
	Unstable - 3 or more fragments^c^	MSP biaxial	
	Unstable - no lateral and/or no posterior support^c^	MSP uniaxial (locked)	
	Stable^d^		DHS
	Intertrochanteric comminution and/or subtrochanteric extension^d^		IMN
Subtrochanteric		MSP uniaxial (locked)	IMN
	Fracture extension below >2 of	IMN or	
	MSP plate screws^e^	long DHS	

^a^Medoff sliding plate (MSP) / dynamic hip screw (DHS)

^b^Intramedullary nail (IMN) / dynamic hip screw (DHS)

^c^Choice of treatment based on Jensen Michaelsen and Seinsheimer classifications including analysis of lateral and posterior support

^d^Choice of treatment based on intertrochanteric fracture comminution or subtrochanteric fracture extension

^e^Approximately 10 cm below the lesser trochanter

From the mutual regional orthopedic surgery database all orthopedic operations performed in the two Skåne regional hospitals during 2011 and 2012 were assessed. Data on all operations with a surgical procedure code for plate or intramedullary nail, and any of the International Code of Disease (ICD-10) codes S7200/01 (femoral neck fracture), S7210/11 (pertrochanteric fracture) or S7220/21 (subtrochanteric fracture), were retrieved (*n* = 892). The total number of previously performed trochanteric hip fracture surgeries was also derived from the database for each individual non-specialist surgeon.

**Fig. 1:**
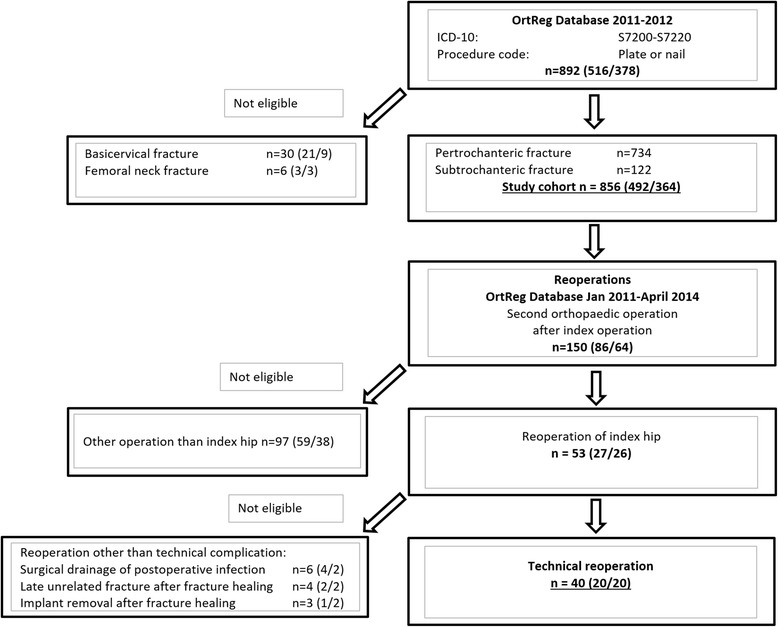
Flow chart depicting the collection of the final study cohort (*n* = 856) and all technical reoperations (*n* = 40) with numbers given in brackets for treatment protocol: Medoff sliding plate/dynamic hip screw (MSP/DHS) followed by treatment protocol: intramedullary nail/dynamic hip screw (IMN/DHS)

All fractures were classified by evaluation of available pre-, intra- and postoperative radiographs. Following exclusion of femoral neck and basicervical fractures, a final study group of 856 patients was set (Fig. 1).

Pertrochanteric fractures were classified according to Jensen Michaelsen (JM) [9], and subtrochanteric fractures according to Seinsheimer (SH) [10]. In the study analysis JM types 1–2 (2 fragments) were considered as stable and JM types 3–4-5 (3 or more fragments) and all subtrochanteric fractures as unstable.

Fractures were also classified regarding lateral and/or posterior support, defined as the presence of intact cortical bone of the femoral shaft at the level of or above the center of the lag screw entry hole.

Fracture classification was performed by a single examiner. To assess interobserver variability 75 randomly selected fractures were also classified by a second independent observer.

To find relevant reoperations all additional orthopedic operations performed in any Skåne regional hospital after the index operation on the 856 study patients between January 2011 and April 2014, thus with a follow-up time span from 16 to 40 months, were derived from the regional database. After exclusion of other orthopedic surgical procedures and of reoperations other than technical, a final total of 40 technical reoperations was set (Fig. 1). For these cases, technical reoperations and the postoperative course of events were analysed in detail using all available postoperative radiographs and medical charts. Quality of fracture reduction and implant positioning was classified as good, acceptable or poor [11–13] and definitions were set to analyse reoperations, final outcome and related technical complications in the four categories adjustment (removal of locking screw, no technical complication) or minor (percutaneous withdrawal, replacement or removal of Twin Hook/lag screw due to penetration, followed by fracture healing), intermediate (reosteosynthesis due to non-union, femoral shaft stress fracture or varisation/cut-out) or major (loss of hip joint integrity with Girdlestone procedure or hip joint replacement, or persistent nonunion).

### Statistical analysis

Statistical analyses were made using Analyse-it Standard Edition 3.20 (Analyse-it Software, Ltd. Leeds, UK). Median was given with 25th and 75th percentiles. Comparisons between groups were made using the chi-squared test or by calculation of relative risk (RR) with 95% C.I. Results with a *p* < 0.05 were considered as significant.

## Results

### Study group

The treatment protocol: MSP/DHS (*n* = 492) and treatment protocol: IMN/DHS (*n* = 364) groups were similar regarding age, gender, distribution of different fracture subtypes, number of surgeons and surgeons experience. Median age was 85 (79–89) years and two thirds of the patients were female. The operations were made by a surgeon having performed 25 or more previous trochanteric hip fracture surgeries in 68% of the patients in treatment protocol MSP/DHS and in 87% in treatment protocol IMN/DHS, respectively. 16% of all fractures were classified as stable pertrochanteric, 70% unstable pertrochanteric and 14% subtrochanteric. Lack of lateral support was noted in 19% and of posterior support in 48% of all fractures.

In assessment of interobserver variability, classification agreement was 75% (56/75) for specific JM/SH fracture subtypes, whereas 97% (73/75) were uniformly classified as stable or unstable.

### Fixation methods

A vast majority of stable pertrochanteric fractures were treated using DHS in both treatment groups. Unstable pertrochanteric fractures, according to study fracture classification, were operated with MSP in biaxial mode (82%) or in uniaxial mode (9%) in treatment protocol: MSP/DHS. In 9% DHS was used, not compliant with the treatment protocol. In treatment protocol: IMN/DHS 35% were operated with IMN and 65% with DHS, but of all fractures JM types 3–4-5 only 2% (6/251) had lack of lateral support and were treated with DHS, contradicting the treatment protocol. Subtrochanteric fractures were treated using IMN (92%) or DHS (8%) in treatment protocol: IMN/DHS, and with MSP in uniaxial (61%) or biaxial mode (7%), with IMN (23%) or with DHS (9%) in treatment protocol: MSP/DHS (Table 2).

**Table 2:** Number of fractures, and fraction within fracture type group, treated with each fixation method for different fracture subtypes in the 2 treatment groups

Fracture type^c^	Treatment protocol: MSP/DHS^a^				Treatment protocol: IMN/DHS^b^	
	DHS	MSP biaxial	MSP locked uniaxial	IMN	DHS	IMN
Stable pertrochanteric	56 (77%)	17 (23%)	0	0	59 (98%)	1 (2%)
JM1	28 (88%)	4 (12%)			39 (98%)	1 (2%)
JM2	28 (68%)	13 (32%)			20 (100%)	0
Unstable pertrochanteric	30 (9%)	288 (82%)	32 (9%)	0	163 (65%)	88 (35%)
JM3	13 (16%)	60 (74%)	8 (10%)		67 (89%)	8 (11%)
JM4	12 (20%)	43 (73%)	4 (7%)		13 (59%)	9 (41%)
JM5	5 (2%)	185 (88%)	20 (10%)		83 (54%)	71 (46%)
Subtrochanteric	6 (9%)	5 (7%)	42 (61%)	16 (23%)	4 (8%)	49 (92%)
Divergence from treatment protocol^d^						
JM1-JM2 with no lateral or no posterior support or JM3-JM4-JM5	31 (1^e^)					
JM 3–4-5 with no lateral support					6 (2^e^)	
JM3-JM4-JM5 with no lateral or no posterior support or subtrochanteric		8 (0^e^)				

^a^Treatment protocol: Medoff sliding plate (MSP)/dynamic hip screw (DHS)

^b^Treatment protocol: Intramedullary nail (IMN)/dynamic hip screw (DHS)

^c^ *JM* Jensen Michaelsen fracture classification

^d^The number of operations among the above with fixation methods with main divergence from the respective treatment protocol for grouped fracture subtypes

^e^Among these, number of reoperations with technical complication

### Treatment groups and reoperations

**Fig. 2:**
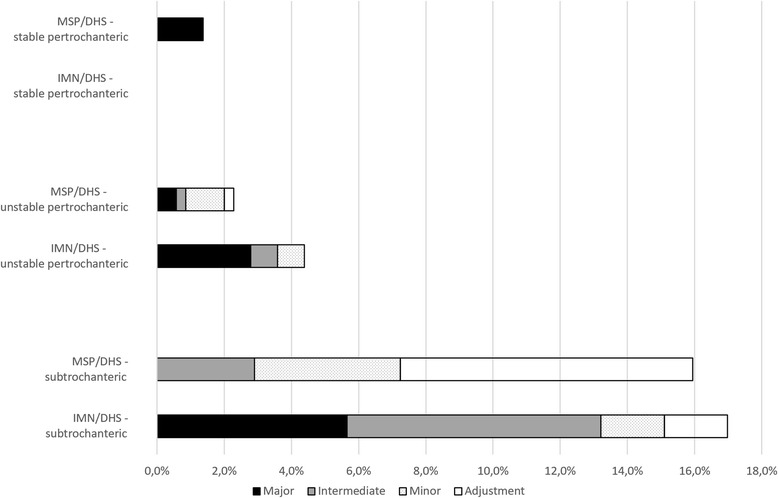
Rates of adjustment reoperations and the different categories of reoperations with technical complications for stable pertrochanteric, unstable pertrochanteric and subtrochanteric fractures in treatment protocol: Medoff sliding plate/dynamic hip screw (MSP/DHS) and in treatment protocol: intramedullary nail/dynamic hip screw (IMN/DHS)

There was a total of 40 technical reoperations in 856 patients, 20 in treatment protocol: MSP/DHS (4.1%, Table 3) and 20 (5.5%) in treatment protocol: IMN/DHS (*p* = 0.33, Table 4). Quality of fracture reduction and of implant positioning in the 40 reoperated patients appeared to be similar in the 2 treatment groups (Tables 3 and 4). 13 technical complications (2.6%) occurred in treatment protocol: MSP/DHS and 19 (5.2%) in treatment protocol: IMN/DHS (*p* = 0.049). The rate of intermediate and major complications was more than 3 times higher (RR 3.6, 1.5–8.9) in treatment protocol: IMN/DHS (16/364, 4.4%) than in treatment protocol: MSP/DHS (6/492, 1.2%) (*p* = 0.0037). Documented absence of fracture healing (joint replacement, Girdlestone procedure or persisting nonunion) occurred in 10 cases in treatment protocol: IMN/DHS (2.7%) and in 3 cases (0.61%) in treatment protocol: MSP/DHS (*p* = 0.011, RR = 4.5, 1.3–15) (Fig. 2).

**Table 3:** Details of all reoperations (*n* = 20) and analysis of mechanisms of technical complications in treatment protocol: Medoff sliding plate/dynamic hip screw

Fracture type	Fixation	Analysis of mechanism	Reflection on mechanism of complication	Reoperation	Technical complication category	Surgeon’s experience^a^	Fracture reduction	Twin hook/lag screw position	Outcome^b^
JM5	MSP uniaxial	Sliding plate consumed	In line with treatment protocol	Locking screw removed	No; adjustment	2	Acceptable	Good	Fracture healing
SH2C						3			
SH3A						4	Good		
SH5						1			
SH5						2	Acceptable		
SH5						3			
SH5						2	Poor		
JM5	MSP biaxial	Intraoperative error -TH wing in hip joint	Avoidable	TH replaced	Minor	3	Poor	To distal	Fracture healing
JM5	MSP uniaxial	TH penetration – sliding plate consumed, but locking screw not removed	Locking screw should have been removed postoperatively, since plate sliding capacity was consumed	TH replaced locking screw removed	Minor	2	Poor	Good	Fracture healing
JM5						4	Good		
SH2C						2			
SH5						3			
SH3A						3	Acceptable		
SH3A						4			
JM3	DHS	Medialisation and varus dislocation	DHS allowed anteromedialisation due to lack of lateral/posterior support	Hip joint replacement	Major	1	Good	Good	Hip joint replacement
JM5	MSP biaxial	TH penetration –medialisation	MSP should have been locked. Biaxial MSP allowed anteromedialisation due to lack of lateral/posterior support	MSP biaxial replaced with MSP uniaxial	Intermediate	3	Acceptable	Good	Fracture healing
JM5		Medialisation and varus dislocation		Hip joint replacement	Major	4	Good		Hip joint replacement
JM2	DHS	Varus dislocation and cut-out	Fracture impaction occured - DHS load-sharing was insufficient with unloading of fracture site	Hip joint replacement	Major	2	Good	Posterior	Hip joint replacement
SH5	IMN	Nonunion with broken IMN	IMN was load-bearing with unloading of fracture site and with stress concentration	IMN replaced with uniaxial MSP	Intermediate	4	Acceptable	Good	Fracture healing

*JM* Jensen Michaelsen fracture classification, *SH* Seinsheimer fracture classification

*TH* Twin hook, *DHS* dynamic hip screw, *MSP* Medoff sliding plate, *IMN* intramedullary nail

^a^Documented number of previous trochanteric hip fracture surgeries performed by index operation surgeon: Less than 10^1^, 10 to 24^2^, 25 or more^3^, or specialist orthopaedic surgeon^4^

^b^Fracture considered as healing if no documentation of diversion from fracture healing at final follow-up

**Table 4:** Details of all reoperations (*n* = 20) and analysis of mechanisms of technical complications in treatment protocol: intramedullary nail / dynamic hip screw

Fracture type	Fixation	Analysis of mechanism	Reflection on mechanism of complication	Reoperation	Technical complication category	Surgeon’s experience^a^	Fracture reduction	Twin hook/lag screw position	Outcome^b^
SH2A	IMN	No fracture impaction	IMN was load-bearing with unloading of fracture site	Locking screw removed	No; adjustment	4	Acceptable	Good	Fracture healing
JM5	DHS	Intraoperative error -TH wing in hip joint	Avoidable	TH replaced	Minor	3	Acceptable	Posterior	Fracture healing
JM3	DHS	TH penetration –anteriorisation	DHS allowed anteromedialisation due to lack of lateral/posterior support	TH replaced	Minor	4	Acceptable	Posterior	Fracture healing^c^
JM5		TH penetration – medialisation		DHS/TH replaced with new DHS	Intermediate	4	Good	Good	Fracture healing
SH2C		Medialisation		DHS replaced with IMN		4	Acceptable		
JM3		Nonunion – anteromedialisation		Girdlestone	Major	4	Good		Girdlestone
JM5						3			
JM3	DHS	Varus dislocation and nonunion	Fracture impaction occured - DHS load-sharing was insufficient with unloading of fracture site	Hip joint replacement	Major	4	Good	Good	Hip joint replacement
JM5		Varus dislocation and cut-out				2			
JM4	IMN	Varus dislocation and cut-out	Fracture impaction occured -IMN was loadbearing with unloading of fracture site	Hip joint replacement	Major	4	Acceptable	Posterior	Hip joint replacement
SH5						3		Good	
SH2C	IMN	Nonunion	IMN was load-bearing with unloading of fracture site	IMN replaced with long DHS	Intermediate	4	Acceptable	Good	Fracture healing
JM5			Removal of IMN and lag screw	Major		4	Good	Posterior	Nonunion
JM5			Hip joint replacement	Major		4		Good	Hip joint replacement
SH3A						4			
SH3A						4	Poor	Proximal	
SH3A	IMN	Broken locking screw	IMN was load-bearing with unloading of fracture site and with stress concentration	IMN locking screw removed	Minor	4	Good	Good	Fracture healing
JM5		Broken IMN		IMN replaced with long IMN	Intermediate	3			
SH5		Femoral shaft stress fracture				4	Poor		
SH4		Nonunion with femoral shaft stress fracture		IMN replaced with long DHS		4			

*JM* Jensen Michaelsen fracture classification, *SH* Seinsheimer fracture classification

*TH* Twin hook, *DHS* dynamic hip screw, *MSP* Medoff sliding plate, *IMN* intramedullary nail

^a^Documented number of previous trochanteric hip fracture surgeries performed by index operation surgeon: Less than 10^1^, 10 to 24^2^, 25 or more^3^, or specialist orthopaedic surgeon^4^

^b^Fracture considered as healing if no documentation of diversion from fracture healing at final follow-up

^c^Recurrence of Twin Hook penetration left in situ

Stable pertrochanteric fractures had a total reoperation rate of 0.75%, unstable pertrochanteric 3.2% and subtrochanteric 16%. Intermediate and major technical complications were more than 4 times more frequent in treatment protocol: IMN/DHS than in treatment protocol: MSP/DHS in both unstable pertrochanteric fractures (*p* = 0.018) and in subtrochanteric fractures (*p* = 0.031, Table 5).

**Table 5:** Number of technical complications in unstable pertrochanteric and in subtrochanteric fractures in the 2 treatment groups

Fracture type	Implant	Treatment protocol		Relative risk^c^
		MSP/DHS^a^	IMN/DHS^b^	
Unstable pertrochanteric		All technical complications		
		7/350 (2,0%)	11/251 (4,4%)	2.9 (0.89–5.4)
	DHS	1/30	7/163	
	MSP biaxial	4/288	-	
	MSP uniaxial	2/32	-	
	IMN	-	4/88	
		Intermediate and major technical complications		
		3/350 (0,86%)	9/251 (3,6%)	4.2 (1.2–14)
	DHS	1/30	5/163	
	MSP biaxial	2/288	-	
	MSP uniaxial	0/32	-	
	IMN	-	4/88	
		Major technical complications		
		2/350 (0,57%)	7/251 (2,8%)	4.9 (1.2–21)
	DHS	1/30	4/163	
	MSP biaxial	1/288	-	
	MSP uniaxial	0/32	-	
	IMN	-	3/88	
Subtrochanteric		All technical complications		
		5/69 (7,2%)	8/53 (15%)	2.1 (0.78–5.8)
	DHS	0/6	1/4	
	MSP biaxial	0/5	-	
	MSP uniaxial	4/42	-	
	IMN	1/16	7/49	
		Intermediate and major technical complications		
		2/69 (2,9%)	7/53 (13%)	4.6 (1.1–19)
	DHS	0/6	1/4	
	MSP biaxial	0/5	-	
	MSP uniaxial	1/42	-	
	IMN	1/16	6/49	
		Major technical complications		
		0/69	3/53 (5,7%)	8.6 (0.45–163)
	DHS	0/6	0/4	
	MSP biaxial	0/5	0	
	MSP uniaxial	0/42	0	
	IMN	0/16	3/49	

*DHS* dynamic hip screw, *MSP* Medoff sliding plate, *IMN* intramedullary nail

^a^Treatment protocol: Medoff sliding plate (MSP)/dynamic hip screw (DHS)

^b^Treatment protocol: intramedullary nail (IMN)/dynamic hip screw (DHS)

^c^95% CI

### Implants and reoperations

**Fig. 3:**
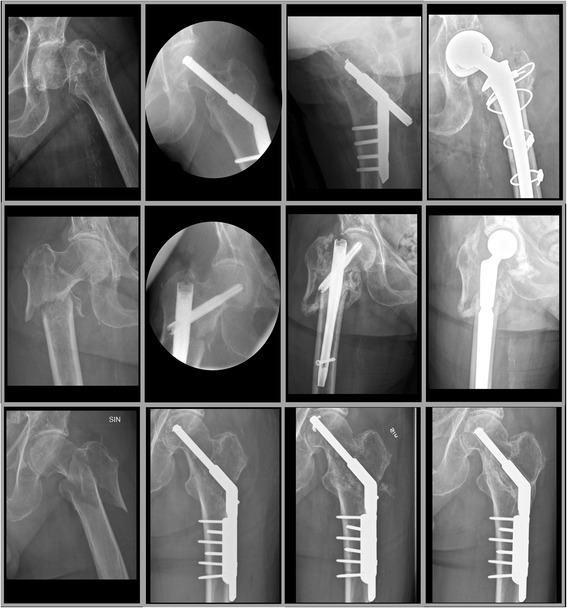
From left to right preoperative, intra/postoperative, post technical complication and final radiographs of the typical and most frequent type of technical complication that occurred with dynamic hip screw (medialisation and nonunion followed by hip joint replacement, upper panels), intramedullary nail (nonunion followed by hip joint replacement, middle panels) and uniaxial Medoff sliding plate (Twin hook penetration followed by Twin hook replacement and fracture healing, lower panels)

Total reoperation rate with biaxial MSP was 1.3%, with DHS 3.1% and with IMN 8.4% (Fig. 3). With MSP in uniaxial mode 13 of 74 (18%) were reoperated, 92% of these were adjustments (*n* = 7) or minor technical complications (*n* = 5). Overall rate of intermediate and major technical complications in all unstable pertrochanteric and subtrochanteric fractures was with uniaxial MSP 1.4% and was lower with biaxial MSP (0.68%) than with IMN (7.2%, *p* = 0.0001) and with DHS (3.4%, *p* = 0.023). For unstable pertrochanteric fractures separately rate of intermediate and major technical complications was lower with biaxial MSP (0.69%) than with IMN (4.5%, *p* = 0.011), and was with DHS 3.1% and with uniaxial MSP none (Table 5). In 42 subtrochanteric fractures operated with uniaxial MSP one intermediate technical complication (2.4%) occurred and in 65 operated with IMN 7 intermediate or major technical complications occurred (11%, *p* = 0.11).

### Lateral/posterior support

In total, 5 reoperations (1.6%) were performed in 308 unstable pertrochanteric fractures with intact lateral and posterior support. Of the 4 technical complications 3 were minor and only one (0.32%) was major. In the 293 unstable pertrochanteric fractures with lack of lateral or posterior support 14 (4.8%) reoperations and technical complications occurred, 11 (3.8%) of these intermediate (*n* = 3) or major (*n* = 8, 2.7%). Lack of posterior support was more frequent than lack of lateral support, but only one of the 14 reoperations had intact posterior support compared to 10 with intact lateral support.

### Implants and lateral/posterior support

In unstable pertrochanteric fractures with intact lateral and posterior support, reoperation rates were with DHS 2/136 (1.5%), with biaxial MSP 1/143 (0.70%) and none of the 24 fractures operated with IM. If either lateral or posterior support also was lacking, 6 (11%) technical complications occurred with DHS, 5 of these intermediate (*n* = 1) or major (*n* = 4, 7.0%) and with biaxial MSP 2.1% (3/145, *p* = 0.0063), one intermediate and one (0.70%) major. All reoperations with IMN in unstable pertrochanteric fractures (4/88, 4.5%) lacked lateral or posterior support.

## Discussion

The major finding in this study was the more than 4 times higher rate of technical complications with loss of hip integrity, persistent non-union or need for revision osteosynthesis, following treatment of both unstable pertrochanteric and subtrochanteric fractures according to treatment protocol: IMN/DHS compared to treatment protocol: MSP/DHS. The low technical complication rate with MSP in both unstable pertrochanteric and subtrochanteric fractures is in line with results reported in previous studies [4, 12, 14–16]. The main mechanisms behind major technical complications were malalignment with varus dislocation and cut-out due to anteromedialisation of the femoral shaft in fractures without lateral or posterior support operated with DHS or biaxial MSP, varus dislocation and cut-out in fractures treated with DHS or IMN due to insufficient load-sharing, or bone or implant stress fracture or nonunion due to load-bearing in fractures treated with IMN.

The retrospective study design was observational using post-hoc analysis of existing data, which usually provides less solid information due to limited control of systematic and consecutive study inclusion and incomplete outcome data. However, the regional orthopedic surgery database structure warranted complete consecutive inclusion of all operated patients as well as complete registration of the sole outcome parameter reoperation. Therefore, the study design may actually be considered a strength, since the objective of studying treatment protocol performance in regular daily care was not biased by study design, or by awareness among involved surgeons of an ongoing study. Systematic repeat radiographs after the postoperative examination were obtained only when uniaxial MSP was used. Reported rates of technical complications for the treatment protocols should therefore be considered as lower limits of true rates.

Assumed treatment group similarity to allow group comparisons appeared justified, since all fractures were consecutively included and no relevant group differences were observed regarding patient baseline data, fracture type distribution or surgical performance. Still, in addition to implants, existence of other group differing factors with possible impact on outcome, such as fracture pattern distribution or hospital specific performance, cannot be completely ruled out. Classification reliability was assessed by interobserver comparison and agreement was high, in particular in the important distinction between stable and unstable fractures.

Similar to other reports [2, 4, 14], the main technical complications with DHS in unstable pertrochanteric fractures were varus dislocation with lag screw cut-out or nonunion, frequently related to femoral shaft medialisation. With MSP in biaxial mode, lower rate of fixation failure in unstable pertrochanteric fractures was reproduced in this study. The sliding mechanism of both the DHS and the MSP lag screw barrel is aligned parallel to the femoral neck. Hence, compression along this axis will induce anteromedial translation of the femoral shaft relative to the head and neck fragment, which may compromise stable contact and load transfer between the main proximal and distal fracture fragments. A sevenfold increase of technical fixation failures with DHS has been reported with a medialisation of 30% or more [17]. As with DHS, the main mechanism behind major complications with biaxial MSP is femoral shaft anteromedialisation, which however appeared to be less frequent and less pronounced than with DHS in this study as well as in others [4, 16], probably due to facilitated axial fracture impaction and interdigitation of fracture fragments. Still, as with DHS, biaxial MSP may be inappropriate if the fracture extends below the level of the lag screw entry hole with subsequent loss of lateral and or posterior support. Fracture malalignment has severe consequences and the vast majority of reoperations related to femoral shaft anteromedialisation in this study were associated with major complications. According to treatment protocol: MSP/DHS MSP should be used in uniaxial mode in the absence of lateral or posterior support. This strategy prompted some adjustment procedures to allow secondary dynamisation, or minor reoperations due to Twin hook/lag screw penetration, but no major complications occurred.

The IMN maintains alignment and prevents femoral shaft anteromedialisation. Still, due to poor load transfer from implant to fracture site, varus dislocation and cut-out may occur following loss of fixation purchase in the femoral head and neck fragment [13, 18]. If purchase is maintained, nonunion, implant breakage or femoral shaft stress fracture may occur due to persistent unloading of the fracture surfaces [19–22], typical complications that were all observed in the present study.

In subtrochanteric fractures lateral and/or posterior support is by definition always absent. Miedel [23] reported reoperations in subtrochanteric fractures operated with MSP due to medialisation. However, in this study the MSP was used in biaxial mode. In subtrochanteric fractures MSP should always be used in uniaxial mode [24, 25] to achieve efficient load-sharing and prevent malalignment, at the expense of need for secondary dynamisation in a minority of patients to avoid risk of Twin hook/lag screw penetration. Nevertheless, as noted in this and in other studies [26, 27], with MSP in uniaxial mode Twin hook/lag screw penetration should not be expected to be completely avoided. With maintained axial and varus/valgus alignment this complication is however less demanding and may be resolved with a minor procedure, particularly with the Twin hook allowing percutaneous adjustments. Compared to the MSP, the IMN had a higher rate of intermediate and major technical complications, all related to load-bearing, when used in subtrochanteric fractures.

In treatment protocol: MSP/DHS the aim was to use MSP in all cases with identification of one or more separate trochanteric fragment, that is all fractures types JM 3–4-5. In treatment protocol: IMN/DHS on the other hand, most of the intermediate unstable pertrochanteric fractures, retrospectively classified as JM3–4-5, but with intact lateral and posterior support were, in accordance with the treatment protocol, treated with DHS. Thus, as opposed to in treatment protocol: MSP/DHS, almost two thirds of pertrochanteric fractures with more than 2 main fragments were operated with DHS. The remaining third operated with IMN therefore most likely represents more complex fractures within these subgroups and implant specific complication rates cannot be directly compared in fractures with the same postoperative classification in the two treatment groups. Accordingly, group comparisons in this study refer to complete fracture type groups. It should also be pointed out that the treatment protocol: IMN/DHS pattern of chosen fixation methods is representative of the overall Swedish national use of fixation methods for pertrochanteric as well as subtrochanteric fractures [28].

The complication rate with DHS was almost as high as with the IMN, suggesting that treatment of mild to moderately complex unstable pertrochanteric fractures with DHS was not optimal. A more than sevenfold increase of the technical complication rate was observed in unstable pertrochanteric fractures treated with DHS, if in addition lack of lateral or posterior support was noted. Whether the intermediate unstable pertrochanteric fracture category would do better with IMN than with DHS cannot be answered by this study. Anteromedialisation due to lack of lateral/posterior support would be controlled by the IMN, but increasing issues related to load-bearing with stress concentration and non-union should be expected. Shortcomings in fracture classification firmly defining these fracture type subgroups limit the value of available studies, as does the fact that most fractures in this subgroup are probably not treated with IMN.

In treatment protocol: MSP/DHS, a vast majority of unstable pertrochanteric fractures were operated with biaxial MSP, which in terms of minimising risk of reoperation and technical complication appears superior to both alternatives for this spectrum of fracture types. Due to surgeons’ assessment of lack of lateral or posterior support, 9% of unstable pertrochanteric fractures in treatment protocol: MSP/DHS were treated with MSP in uniaxial mode. Among these, 2 lag screw penetrations occurred but no major complications, again supporting priority of alignment control over biaxial dynamisation in appropriate fracture patterns. From analysis of mechanisms of technical complications in this study as well as others [29], fractures at increased risk of reoperation can often be identified if additional parameters such as lateral and posterior support are examined.

Reoperations with minor complications had no documented divergence from final fracture healing and were by definition performed percutaneously. This type of technical complication per se should have minor consequences for most patients. Intermediate and major technical complications should be expected to have more profound consequences and these subgroups comprise the cases that should be considered true failures from a technical orthopedic surgical point of view.

## Conclusions

In summary, in this consecutive retrospective observational study comparing two different treatment protocols, technical complications in unstable pertrochanteric and subtrochanteric fractures were less frequent and less severe with a treatment protocol based on MSP fixation, compared to a treatment protocol based on IMN fixation. In this respect, a vast majority of unstable pertrochanteric fractures are best treated with MSP in biaxial mode and stand greater risk of major complications if treated with DHS, due to loss of alignment or insufficient load-sharing, or with IMN due to load-bearing. In the most challenging unstable pertrochanteric and subtrochanteric fractures proper application of the MSP in uniaxial mode, in combining maintained alignment and effective load-sharing, will minimise the risk of major technical complications compared to DHS and IMN.

## Abbreviations

DHS: Dynamic hip screw
IMN: Intramedullary nail
JM: Jensen & Michaelsen classification
MSP: Medoff sliding plate
SH: Seinsheimer classification
TH: Twin hook
